# Lung responses to secondary endotoxin challenge in rats exposed to pig barn air

**DOI:** 10.1186/1745-6673-3-24

**Published:** 2008-10-30

**Authors:** Chandrashekhar Charavaryamath, Taryn Keet, Gurpreet K Aulakh, Hugh GG Townsend, Baljit Singh

**Affiliations:** 1 Immunology and Infectious Disease Research Group, University of Saskatchewan, Saskatoon, SK S7N 5B4, Canada; 2 Department of Veterinary Biomedical Sciences, University of Saskatchewan, Saskatoon, SK S7N 5B4, Canada; 3 Department of Large Animal Clinical Sciences, University of Saskatchewan, Saskatoon, SK S7N 5B4, Canada

## Abstract

**Background:**

Swine barn air contains endotoxin and many other noxious agents. Single or multiple exposures to pig barn air induces lung inflammation and loss of lung function. However, we do not know the effect of exposure to pig barn air on inflammatory response in the lungs following a secondary infection. Therefore, we tested a hypothesis that single or multiple exposures to barn air will result in exaggerated lung inflammation in response to a secondary insult with *Escherichia coli *LPS (*E. coli *LPS).

**Methods:**

We exposed Sprague-Dawley rats to ambient (N = 12) or swine barn air (N = 24) for one or five days and then half (N = 6/group) of these rats received intravenous *E. coli *LPS challenge, observed for six hours and then euthanized to collect lung tissues for histology, immunohistochemistry and ELISA to assess lung inflammation.

**Results:**

Compared to controls, histological signs of lung inflammation were evident in barn exposed rat lungs. Rats exposed to barn air for one or five days and challenged with *E. coli *LPS showed increased recruitment of granulocytes compared to those exposed only to the barn. Control, one and five day barn exposed rats that were challenged with *E. coli *LPS showed higher levels of IL-1β in the lungs compared to respective groups not challenged with *E. coli *LPS. The levels of TNF-α in the lungs did not differ among any of the groups. Control rats without *E. coli *LPS challenge showed higher levels of TGF-β2 compared to controls challenged with *E. coli *LPS.

**Conclusion:**

These results show that lungs of rats exposed to pig barn air retain the ability to respond to *E. coli *LPS challenge.

## Background

Swine production is a major agricultural industry in Canada and employs many fulltime workers who may work in shifts of 8 hours/day and 5 days/week inside the confined barns (reviewed in [1]). Full-time barn workers experience multiple-interrupted exposures to complex swine barn environment [2-5]. Swine barn environment is a heterogeneous mixture containing organic dust, various microbes, endotoxin and a number of gases such as ammonia, carbon dioxide, hydrogen sulphide and methane [2,6,7]. Therefore, despite clean appearance, the modern large scale barns pose greater health risk to swine barn workers [8].

Exposure to barn air causes respiratory symptoms, loss of lung function, increased airway hyperresponsivneess (AHR) and airway inflammation (reviewed in [1]). Single (2–5 hour) experimental exposure of naïve human volunteers to barn environment induces fever, malaise, drowsiness [9], bronchial responsiveness [10] and lung inflammation with increased influx of neutrophils, lymphocytes, eosinophils and macrophages in broncholavelolar lavage fluid (BALF) as well as chemoattractants such as IL-8 [8,9,11]. When compared to naïve volunteers, repeatedly exposed swine farmers demonstrate accentuated inflammatory and airway responses following a single experimental barn exposure [9,12,13] to indicate a possible adaptation response.

Recently, we have used rat and mouse models to mimic occupational exposures of full-time barn workers and demonstrate that single or five exposures to the barn air induce lung inflammation and AHR. Interestingly, the responses were attenuated after 20 exposures to the barn [14,15]. We have also reported that barn air induced lung inflammation but not AHR is dependent on TLR4 activation [15]. Recently, we showed transient recruitment of pulmonary intravascular monocytes/macrophages (PIMMs) in rats at 48 hours after a single 8-hour exposure to the barn air and that treatment of these rats with *Escherichia coli *LPS (*E. coli *LPS) at 48 hours after the barn exposure resulted in robust lung inflammation [16]. Taken together, these data showed that single exposure to barn air induced recruitment of PIMMs and recruited PIMMs may mediate exacerbation of lung inflammation in response to a secondary challenge with *E. coli *LPS.

To date, we do not know lung responses of barn exposed animals to secondary challenge with LPS prior to PIMM recruitment. Because there is potential that a barn worker may be exposed to a bacterial infection within a few hours of finishing a work shift, it is important to understand this lung response. Since our previous work has shown significant recruitment of PIMMs at 48 hours post-single barn exposure [16], it is important to understand the host response to a secondary LPS challenge prior to this time point.

Therefore, in the current study, we used a recently characterized rat model of barn air induced lung inflammation to test a hypothesis that lungs of rats exposed to single or multiple times to pig barn air will be competent to respond to a *E. coli *LPS challenge at 18 hour post barn exposure. Our data show that lungs of rats exposed to the barn air remain capable of mounting an effective innate inflammatory response to a secondary *E. coli *LPS challenge.

## Materials and methods

### Rats and treatment groups

The animal experiment protocols were approved by Animal Research Ethics Board, University of Saskatchewan, Saskatoon, Canada and were conducted according to the Canadian Council on Animal Care Guidelines. Specific pathogen-free, six-week-old, male, Sprague-Dawley rats (Charles River Laboratories, Canada) were maintained in the animal care unit of Western College of Veterinary Medicine. Rats were randomly assigned to six groups (n = 6 each). All the personnel involved in collection and analyses of samples were blinded to the treatment groups.

### Exposure to swine barn air and *E. coli *LPS challenge

The barn exposure procedure has been described previously [14]. Briefly, the rats were placed in the cages and the cages were hung from the barn ceiling approximately at a height of two meters from the floor. Rats were exposed either to ambient air (N = 12) or to the barn air (N = 24). Barn exposure was for a period of eight hours per day for one (N = 12) or five days (N = 12). Immediately following exposure to the barn or ambient air, one half of these rats (n = 6/group) were euthanized and lung tissues were collected. The remaining half of the rats received a secondary challenge with *E. coli *LPS intravenously (1.5 μg/kg of body weight, Sigma-Aldrich, MD) 18 hours after completion of the barn exposure, observed for six-hours and then euthanized prior to collection of lung tissues for histology, immunohistochemistry and ELISA. Previously, we demonstrated induction of lung inflammation following intravenous administration of *E. coli *LPS (1.5 μg/kg) [16,17].

### Tissue collection and processing

Lung tissues were collected and processed as described previously [14,17]. Briefly, following euthanasia, three pieces from each lung lobe (left and right) were taken and fixed in 4% buffered-paraformaldehyde for 16–18 hours and embedded in paraffin. Haematoxylin and eosin stained, five micron thick sections, were used for histopathological evaluation of lung inflammation. Remaining lung tissue was snap frozen in liquid nitrogen and stored at -80°C until used.

Semi-quantitative evaluation of lung inflammation was performed as described before [15]. Briefly, histological signs of lung inflammation, such as perivascular and peribronchiolar inflammation as well as perivascular edema, were evaluated by an observer blinded to the study design. Stained slides were coded and randomly selected fields (40 × objective covering an area of 0.096 mm^2^/field) were used for subjective grading of histological changes. Absence of inflammation and edema was recorded as, "-", minimal inflammation as, "+", moderate as, "+ +", intense as, "+ + +" and very intense as, "+ + + +". When intensity of inflammation was intermediate between two successive grades such as "-"and "+", a range, "- to +" was assigned.

### Immunohistochemistry

Lung sections were processed for immunohistochemistry as described [18]. Briefly, the sections were deparaffinized, hydrated and incubated with 5% hydrogen peroxide for 30 minutes to quench endogenous peroxidase, treated with pepsin (2 mg/ml in 0.01 N HCl) for 45 minutes to unmask the antigens and blocked with 1% bovine serum albumin for 30 minutes. Sections were incubated with primary antibodies against TNF-α(1:50), IL-1β(1:25), TGF-β2 (1:100) (all from Santa Cruz Biotechnology, Inc., CA), ED-1 (1:150, mouse anti rat CD68, AbD Serotec, NC) and anti-granulocytes (1:50, BD Biosciences, Mississauga, ON, Canada) followed by horseradish peroxidase (HRP)-conjugated respective secondary antibodies (1:150; DAKO A/S, Denmark). The reaction was visualized using a colour development kit (VECTOR -VIP, Vector laboratories, USA). Controls consisted of staining without primary antibody or with isotype matched immunoglobulin instead of primary antibody.

We used ED-1 and anti-granulocyte antibodies to detect and quantify septal macrophages and granulocytes in the lungs respectively. Previously, ED-1 antibody has been shown to recognize a lysosomal protein in rat monocytes/macrophages [19,20], while anti-granulocyte antibody recognizes all types of granulocytes [21] and has previously been used by our group [16]. Following immunohistochemistry, stained slides (n = 3/group) were coded and twenty randomly selected fields (High Power Field (HPF) covered by 40 × objective) were used for counting ED-1 and anti-granulocyte positive cells in the lung septae.

### Enzyme-Linked Immunosorbent Assay (ELISA)

We followed sandwich ELISA protocols to measure the concentrations of TNF-α, IL-1β and TGF-β2 using commercially available capture/detection antibody pairs and recombinant protein standards (TNF-α, BD Biosciences, ON, Canada and IL-1β and TGF-β2, R&D Systems, MN, USA) as described before [15,17,22]. Briefly, lung samples were homogenized in Hanks balanced salt solution (HBSS) (100 mg lung tissue/ml of HBSS) containing protease inhibitor cocktail (100 μl/10 ml; Sigma-Aldrich, St. Louis, MO, USA). ELISA plates were coated with capture antibody (over night at 4°C), blocked with 1% bovine serum albumin (Sigma Aldrich, Canada) followed by addition of standards and samples (n = 3,100 μl each in duplicates) and incubation over night at 4°C. The plates were washed with PBS-Tween and incubated with detection antibody (60 minutes at 37°C) followed by color detection reagents and reading at 450 nm.

### Statistical analyses

All data were expressed as mean ± SD. Group differences were examined for significance using two-way analysis of variance with Tukey Test as *post hoc *test (SigmaStat for Windows Version 3.11, San Jose, CA). Significance was established at P < 0.05.

## Results

### Histopathology of lung sections

**Figure 1:**
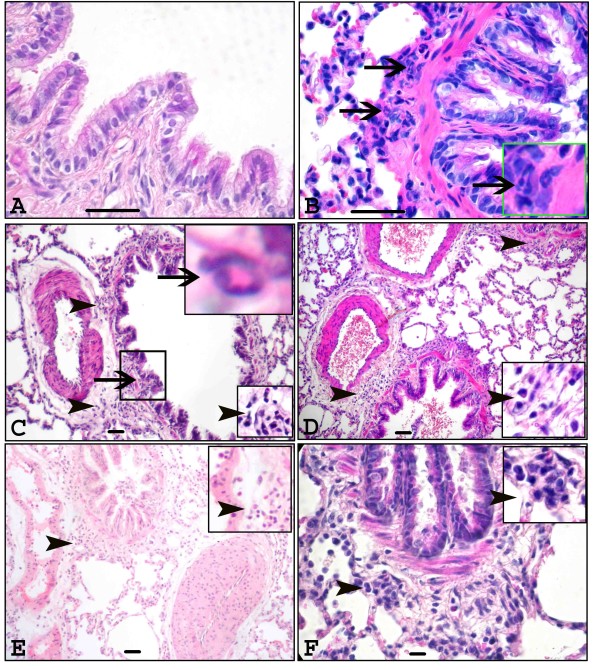
**Histopathology of lung sections**. Histopathological changes in the lungs of rats exposed either to ambient (control) or swine barn air with or without *E. coli *LPS challenge were evaluated using hematoxylin and eosin stained sections. Control rat lung tissues showed no inflammation and normal architecture of the organ (A) while rats challenged with *E. coli *LPS (B), one day barn exposed rats without *E. coli *LPS (C) and with *E. coli *LPS (D), five day barn exposed rats with or without *E. coli *LPS (E and F respectively) showed peribronchiolar (arrows and inset, B) and septal neutophilic infiltration (arrows and inset, C), perivascular infiltration of leukocytes (arrowheads and insets, C-E), and peribronchiolar accumulation of leukocytes (arrowhead and inset, F). *Original magnification *A-B: ×400, C-F: ×100 and micrometer bar = 50 μm.

Semi quantitative evaluation of histological signs of lung inflammation is summarized in Table 1. Control rat lungs showed no signs of inflammation (Figure 1A) while rats treated with intravenous *E. coli *LPS alone and one or five day barn exposed rats with or without *E. coli *LPS treatment showed lung inflammation characterized by peribrochiolar infiltration of neutrophils (Figure 1B), perivascular and peribrochiolar infiltration of inflammatory cells (Figure 1C–F) and perivascular edema (picture not shown).

**Table 1:** Semi-quantitative evaluation of histological inflammation in lung sections.

**Treatment groups**	**Peri-vascular inflammation**	**Peri-bronchiolar inflammation**	**Peri-vascular edema**
Control	- to +	- to +	- to+
Control+LPS	+ + to + + +	+ + to+ + +	+ to+ +
1 day exposure	+ + to+ + +	+ + to + + + +	+ + + +
1 day exposure+LPS	+ + to + + + +	+ + to + + + +	+ + to + + + +
5 day exposure	+ to + + +	+ to + + +	+ + to + + +
5 day exposure + LPS	+ + to + + +	+ +	+ + to + + +

### Immunohistochemistical identification and quantification of macrophages and granulocytes

**Figure 2:**
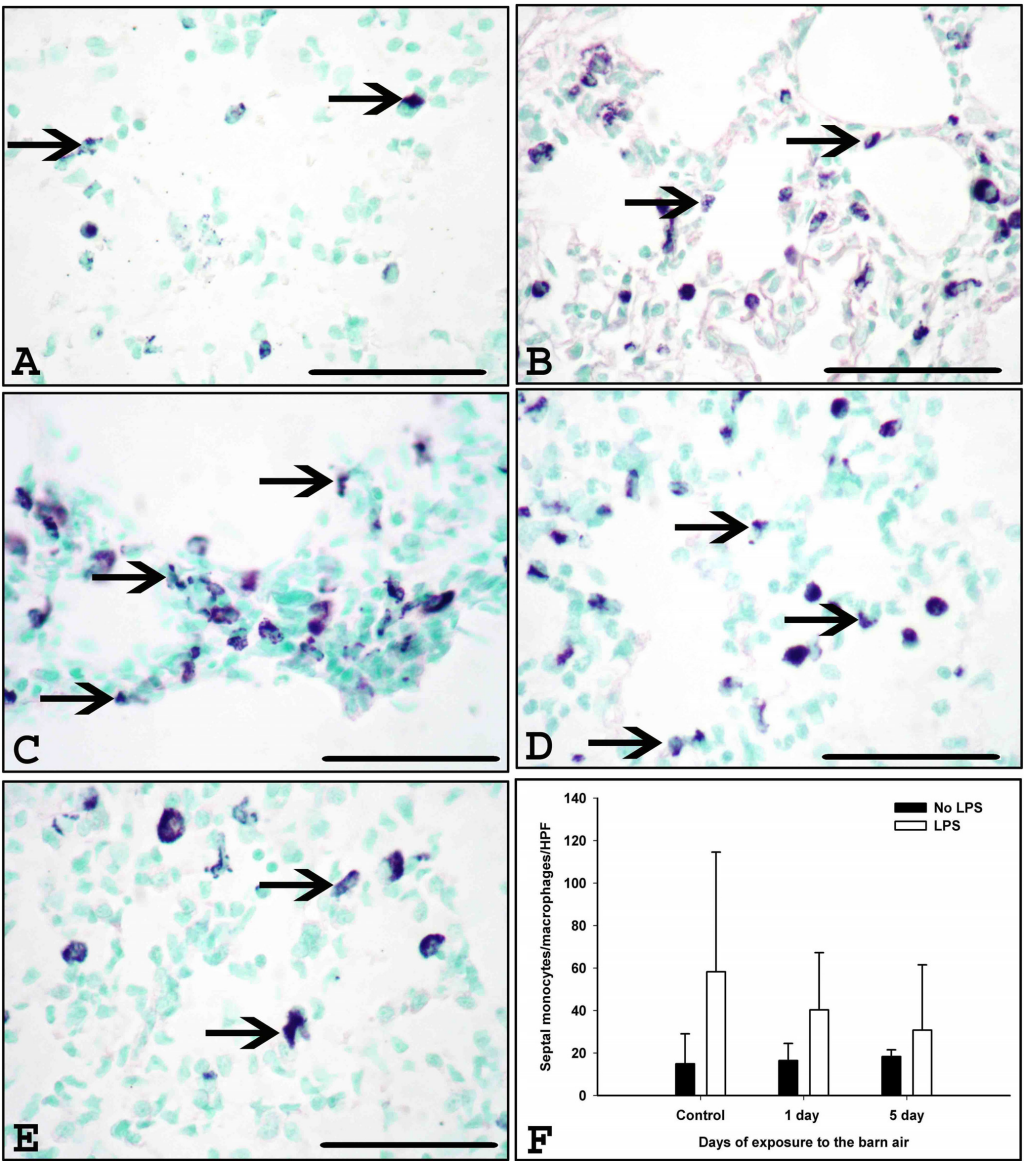
**Immunohistochemical quantification of monocytes/macrophages in the lung**. Monocytes/macrophages were stained using ED-1 antibody in the lung sections from control (A), *E. coli *LPS (B), one day barn exposed rats without and with *E. coli *LPS challenge (C-D) and five day barn exposed rats without (E) (arrows) and with *E. coli *LPS challenge respectively (picture not shown) F: Quantification of septal monocytes/macrophages revealed no significant difference among any of the groups (P > 0.05).*Original magnification *A-F: ×400 and micrometer bar = 50 μm.

**Figure 3:**
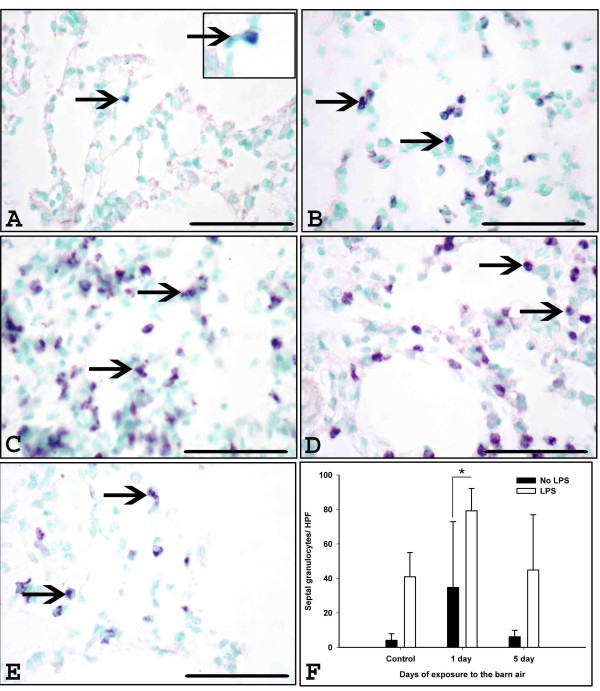
**Immunohistochemical quantification of granulocytes in the lung**. Granulocytes in the lung sections were stained using anti-granulocyte antibody from control (A, arrows and inset), *E. coli *LPS (B), one day (C-D) exposed rats without and with *E. coli *LPS challenge and five day (E) barn exposed rats without (arrows, B-E) and with *E. coli *LPS challenge (picture not shown) respectively. F: Quantification of septal granulocytes showed increased numbers in one day exposed rats with *E. coli *LPS challenge compared to one day exposed rats without *E. coli *LPS challenge (P = 0.029). Five day exposed rats with *E. coli *LPS challenge show a trend towards significant increase when compared to respective five day exposed rats without *E. coli *LPS challenge (F, P = 0.051). *Original magnification *A-F: ×400 and micrometer bar = 50 μm.

There was no significant difference in the mean number of ED-1 positive cells in the lung septae among all the groups (Figure 2F, P > 0.05). The mean number of granulocytes was increased in the lung septae of one (P = 0.029) or five (P = 0.051) day exposed rats challenged with *E. coli *LPS when compared to one or five day exposed rats not treated with the LPS (Figure 3F).

### Expression and quantification of IL-1β

**Figure 4:**
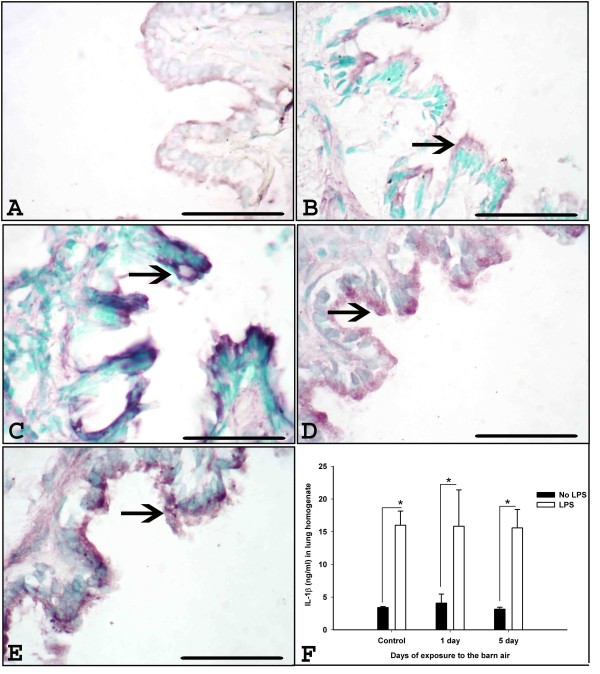
**IL-1β expression and quantification in the lung**. Immunohistochemical expression of IL-1β was detected using anti-IL-1β antibody in the lung sections from controls (A-B), one day (C-D) exposed rats without and with *E. coli *LPS challenge respectively, five day exposed rats without *E. coli *LPS challenge (E) and with *E. coli *LPS challenge (picture not shown). IL-1β expession in the airway epithelium (arrows, A-E) is shown. F. Quantification of IL-1β protein using ELISA shows increased concentrations in rats that received *E. coli *LPS compared to respective groups of rats that did not receive *E. coli *LPS (Figure 4, P < 0.001). *Original magnification *A-F: ×400 and micrometer bar = 50 μm.

Immunohistochemistry detected staining for IL-1β in airway epithelium (Figure 4, A–E), blood vessel wall, lung septa and occasionally in alveolar macrophages (AMs) (data not shown). Quantification with ELISA revealed significantly higher IL-1β concentrations in the lungs of ambient air or barn exposed rats (one or five exposures) that received *E. coli *LPS challenge compared to respective groups without *E. coli *LPS challenge (Figure 4F, P < 0.001).

### Expression and quantification of TNF-α

**Figure 5:**
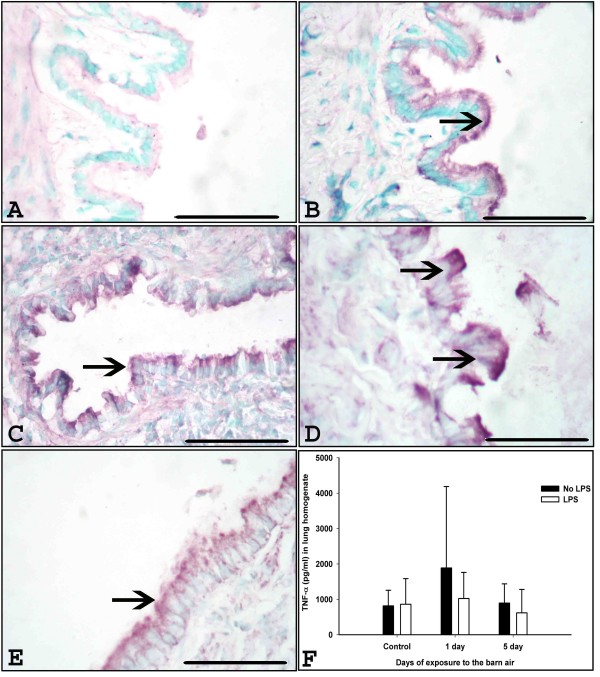
**TNF-α expression and quantification in the lung**. Immunohistochemical expression of TNF-α was detected using anti-TNF-α antibody in the lung sections from controls (A-B), one day (C-D) exposed rats without and with *E. coli *LPS challenge respectively, five day exposed rats without *E. coli *LPS challenge (E) and with *E. coli *LPS challenge (picture not shown). TNF-α expression in the airway epithelium (arrows, A-E) is shown. F. Quantification of TNF-α protein using ELISA showed no significant difference among any of the groups (Figure 5, P > 0.05). *Original magnification *A-F: ×400 and micrometer bar = 50 μm.

Immunohistochemistry detected TNF-α in airway epithelium (Figure 5A–E), blood vessel wall, lung septa and occasionally in AMs and quantification using ELISA revealed no difference among any of the groups (Figure 5F, P > 0.05).

### Expression and quantification of TGF-β2

**Figure 6:**
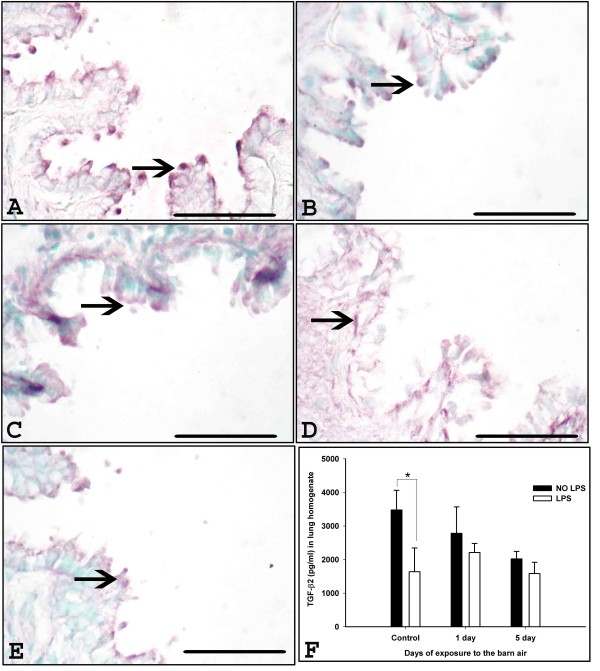
**TGF-β2 expression and quantification in the lung**. Immunohistochemical expression of TGF-β2 was detected using anti- TGF-β2 antibody in the lung sections from controls (A-B), one day (C-D) exposed rats without and with *E. coli *LPS challenge respectively, five day exposed rats without *E. coli *LPS challenge (E) and with *E. coli *LPS challenge (picture not shown). TGF-β2 expression in the airway epithelium (arrows, A-E) is shown. F. Quantification of TGF-β2 protein using ELISA showed increased levels in control rats without *E. coli *LPS challenge compared to control rats with *E. coli *LPS challenge (*, P = 0.001).*Original magnification *A-F: ×400 and micrometer bar = 50 μm.

Immunohistochemistry detected TGF-β2 in airway epithelium (Figure 6A–E), blood vessel wall, lung septa and occasionally in AMs and quantification using ELISA revealed that control rats without *E. coli *LPS challenge showed higher levels of TGF-β2 compared to controls rats challenged with *E. coli *LPS (Figure 6F, P = 0.001).

## Discussion

We conducted this study to investigate lung responses to a secondary LPS challenge in rats exposed to barn air. Our data show that lungs of rats exposed to barn air became more inflamed following challenge with LPS when compared to those exposed to barn air only. These data suggest that lungs of animals exposed to pig barn air are capable of responding to microbial challenges.

We have previously demonstrated that significant PIMM recruitment is observed at 48 hours after a single exposure to pig barn air and these recruited PIMMs exacerbated lung inflammation in response to a secondary LPS challenge [16]. Now, we studied secondary LPS-induced lung inflammation in rats exposed to the barn air prior to PIMM recruitment. We chose to do an *E. coli *LPS challenge at 18 hours after single or five day barn exposure and confirmed the lack of significant recruitment of PIMMs at these time points by immunohistochemical staining using ED-1 antibody which is a known marker for rat monocytes/macrophages.

In the current study we have demonstrated induction of lung inflammation following one or five days of barn exposure as before [14-16]. The *E. coli *LPS challenge of one day barn exposed but not control rats at 18 hour post-exposure showed an increase in granulocyte recruitment compared to respective control groups. However, there were no differences in granulocyte numbers between LPS-treated barn exposed and control animals. Neutrophils are the predominant granulocytes recruited into the inflamed lung [23] and are considered central to development of acute lung inflammation [24,25]. As expected we did not observe an increase in lung monocyte/macrophage numbers at 6 hour post-LPS challenge, as this early time point in acute lung inflammation is characterized by an early recruitment of neutrophils followed by monocytes and macrophages in the later periods [26,27]. However, our previous work in rat endotoxin-induced lung inflammation model has also shown increased monocytes recruitment at 3 and 24 hour time points, both dependent on neutrophils [28]. Our current data show that lungs of animals, that had been exposed to barn air, experienced an inflammatory response following challenge with LPS that was equal to, if not greater than that experienced by animal lungs that had not been exposed to barn air prior to LPS challenge. Endotoxin or LPS in the barn or intravenously administered LPS alone used in this study will likely induce different host responses. Intravenously administered LPS very likely induce systemic vascular activation including lung vascular inflammation and exacerbate lung inflammation [16,17]. In contrast to this, LPS in the barn air is associated with other contaminants in the barn and primarily induces lung inflammation and associated respiratory symptoms [1]. Some of the differences in the host responses following intravenous LPS challenge or the barn exposure may be due to differences in dose/exposure, duration of exposure and presence of other agents in the barn will all account for differences in host responses.

Barn exposed as well as control rats challenged with LPS showed increased concentrations of IL-1β but not TNF-α in lung homogenates compared to their respective controls. Again, there were no differences in IL-1β or TNF-α expression between barn exposed and control rats challenged with LPS. IL-1β is produced by monocytes/macrophages and neutrophils as well as endothelial cells and fibroblasts, and is a known early response cytokine in acute lung inflammation and induces expression of adhesion molecules to regulate neutrophil migration [29-33]. IL-1β also directly activates neutrophils through stimulation of mitogen activated protein kinases to result in increased superoxide anion production and respiratory burst in neutrophils [34,35]. IL-1β also induces fever, increases vascular permeability, production of IL-6 and leukocyte adherence to endothelium [36,37]. On the other hand, neutralization of IL-1β has proven protective and beneficial to the host [38]. Based on the expression of these two proinflammatory cytokines, it appears that single or multiple exposures to barn air do not dampen the inflammatory response of lungs to LPS challenge.

Because lung inflammation is controlled by a complex network of both pro and anti-inflammatory cytokines [39], we examined the tissue expression and quantification of TGF-β2, a known anti-inflammatory cytokine with important roles in tissue repair and remodeling [40,41]. The data show reduced expression of TGF-β2 in LPS challenged control rats compared to control rats without *E. coli *LPS challenge. This observation indicates that the suppression of TGF-β2 in inflamed lungs may be due to an active inflammatory reaction which is similar to previous reports of suppression of TGF-β2 expression in lungs of LPS challenged rats [42]. We have reported similar data from rats challenged with *E. coli *LPS at 48 hours after single barn exposure which contain significant numbers of PIMMs [16]. In contrast to LPS challenge 48 hours after an exposure to barn air or of control rats, we did not observe any change in the expression of TGF-β2 when rats were challenged with LPS at 18 hours after one or five exposures to barn air. It seems that exposure to barn air may have suppressed expression of TGF-β2 to LPS challenge. Considering well established roles of TGF-β2 in lung injury and repair, it may be useful to undertake further studies to explore the role of this cytokine in lung inflammation and repair following exposure to the barn air. Furthermore, because of the involvement of TLR4, it may also be useful to conduct in vitro studies to clarify the cell signaling pathways activated by the barn air.

## Conclusion

Our data show that exposure to swine barn environment for one or five days induces lung inflammation, while a secondary challenge with *E. coli *LPS exacerbates the lung inflammation in rats exposed to pig barn air. These data suggest that exposure to barn air does not suppress lung's ability to respond to a secondary LPS challenge.

## Competing interests

The authors declare that they have no competing interests.

## Authors' contributions

CC carried out the experiment, did the histopathological evaluation, statistical analyses and drafted the manuscript. TK did the immunohistochemistry and GKA participated in the quantification of immunohistochemistry data. HT participated in statistical analyses and manuscript preparation. BS conceived of the study, participated in its design and coordination as well as manuscript preparation. All the authors have read and approved the final manuscript.
